# Clinical outcomes of a joint ICU and palliative care multidisciplinary rounding model: A retrospective cohort study

**DOI:** 10.1371/journal.pone.0297288

**Published:** 2024-02-01

**Authors:** Dov Shalman

**Affiliations:** Department of Geriatric, Palliative, and Continuing Care, Kaiser Permanente Southern California, Los Angeles, California, United States of America; Stamford Health, UNITED STATES

## Abstract

**Objectives:**

This retrospective cohort study assessed whether implementation of a joint inpatient palliative care (IPC) and ICU multidisciplinary rounding model affected clinical outcomes including ICU length of stay (LOS).

**Methods:**

Beginning in October of 2018, an IPC physician joined the pre-existing ICU multidisciplinary rounds. Data were collected for ICU patients admitted during a 6-month period before this intervention and a 6-month period after the intervention. Data were extracted from an integrated electronic medical records (EMR) data system and compared by Wilcoxon and chi-square test for continuous and categorical variables respectively. Negative binomial regression was used to analyze the primary outcome measure, ICU LOS.

**Results:**

Patients in the intervention group spent fewer days in the ICU (3.7 vs. 3.9 days, p = 0.05; RR 0.82, 95% CI 0.70–0.97, p = 0.02) and in the hospital (7.5 vs. 7.8 days, p<0.01) compared to the pre-intervention group. The rate of CPR was lower in the intervention group, but the difference was not statistically significant [13(3.1%) vs. 23(5.3%), p = 0.10]. The groups did not differ significantly in rate of hospital mortality, number of days connected to mechanical ventilation via endotracheal tube, or bounceback to the ED or hospital. Multivariable analysis of the primary outcome demonstrated that patients with prior palliative care involvement had longer ICU LOS (RR 1.46, 95% CI 1.04–2.06, p = 0.03) when controlling for other variables.

**Conclusion:**

The presented joint IPC-ICU multidisciplinary rounding model was associated with a statistically significant reduction in ICU and hospital LOS, but the clinical significance of this reduction is unclear.

## Introduction

### Background

Many patients admitted to the ICU have serious life-limiting illnesses and would benefit from the involvement of an Inpatient Palliative Care (IPC) team. These critically ill patients often have distressing physical symptoms and discomfort associated with ICU procedures such as mechanical ventilation [1–4], and their treatment courses can be overwhelmingly complex and emotionally taxing for them and their loved ones [5–8]. In the ICU setting, an IPC team typically helps with pain and symptom management and helps support patients, their families, and their treatment teams in complex medical decision-making. The most common reason for palliative care consultation in the critical care setting is for clarification of “goals of care,” [9–12] which may include discussions around continuation or discontinuation of mechanical ventilation and other forms of artificial life support. ICU providers have identified communication difficulties with patients/families regarding treatment goals and realistic expectations as a barrier to high quality end-of-life care in the ICU [13]. Similarly, ICU patients and their families identified communication and patient-centered decision-making as key to high-quality ICU care [14]. Lilly et al. developed a more formalized approach to proactive communication with families of patients at high risk of dying and found that the intervention improved consensus within the treatment team and with family; it was also associated with a decrease in ICU length of stay (LOS) and ICU mortality [15, 16].

A recent multicenter study by Cobert et al. found that palliative care consultation on ICU patients has increased over the last decade [12]. However, several studies have found that in the absence of formalized processes for palliative care referral, IPC is an underutilized resource in ICU setting—even in institutions with robust palliative care programs [7–9, 17–20]. Multiple critical care workgroups and societies recommend both improved access to specialty palliative care and better incorporation of palliative care principles in the ICU setting [5, 21, 22]. Access to palliative care resources in ICU improves clinical outcomes, including: increased rates of changing code status to Do Not Resuscitate (DNR) [17, 23–25], symptomatic improvement [23], earlier and increased frequency of family meetings [24, 26], decreased duration of mechanical ventilation [24, 25], decreased rate of tracheostomy [25], and increased referral to hospice [25, 27]. Furthermore, there have been a multitude of studies looking at the utilization-related and financial implications of improving access to palliative care in the ICU. Most of these studies have found that palliative care interventions decrease ICU and/or hospital LOS [17, 24, 26, 28–32] and overall cost of admission [31, 32], without significantly affecting hospital mortality. The few studies demonstrating limited benefit include: a non-randomized case-controlled study comparing groups with dissimilar baseline characteristics [33]; a very large multicenter retrospective study comparing centers with and without palliative care programs, which demonstrated statistically but not clinically significant decreases in hospital and ICU LOS in centers with palliative care programs but did not measure actual utilization of these programs [34]; and a single-center crossover study demonstrating multiple clinical benefits but no decrease in LOS [25].

Earlier involvement of palliative care is key to observing the LOS and cost-saving benefits of palliative care consultation in the ICU setting [31, 32]. While IPC involvement is clearly beneficial to many ICU patients, it can be challenging to identify which patients have palliative care needs and ensure timely IPC involvement in those case. Several models have been examined as potential ways to identify patients with palliative care needs and deliver support to them and their families [24]. Integrative approaches focus on training ICU providers to identify appropriate patients and provide primary palliative care interventions [6, 35–38], whereas consultative models focus on identifying patients to receive formal IPC consultation. Consultative models are more efficacious and less burdensome for ICU providers when compared to integrative models [24]. Many consultative models utilize “triggers” for IPC consultation based either on illness-severity [20, 25, 29, 39–42] or palliative care needs [43], while others involve having a palliative care practitioner actively engage with the ICU team regarding potential consultations [17–19, 26, 29, 44]. While many ICU providers agree that trigger models are a useful tool in improving access to palliative care for their patients, a survey of ICU providers demonstrated that many ICU physicians would want to be involved in development of referral criteria and be responsible for ultimately deciding whether to consult IPC [20]. One significant limitation of traditional illness-severity trigger models is that they likely do not capture all patients with palliative care needs [43], specifically younger patients and those in cardiac/cardiothoracic care units [44].

### Objective

In the current study, we retrospectively explored the impact of a joint IPC and ICU multidisciplinary rounding model, which was implemented in October of 2018. Like the model described by Walker et al. [29], a palliative care physician joined ICU multidisciplinary rounds twice per week to review all patients on the census and recommend IPC consultation when appropriate. While similar studies [26, 29] utilized screening criteria along with joint rounding in order to focus on patients with identifiable palliative needs, the current study explores the impact of the joint rounding model without pre-screening for palliative care needs. We hypothesized that increased palliative care presence in the ICU setting would encourage more proactive serious illness communication in patients both with and without clear palliative care needs, resulting in improved clinical outcomes such as ICU and hospital LOS without significantly affecting hospital mortality.

## Materials and methods

### Setting

This retrospective study focused on outcomes in a cohort of adult patients that were admitted to a 560-bed Kaiser Permanente (KP) tertiary medical center in Southern California before and after implementation of a pilot intervention of a joint IPC and ICU multidisciplinary rounding model in October of 2018. The hospital is not a designated trauma center and almost exclusively serves patients who are insured with Kaiser insurance. It houses two 16-bed medical-surgical ICUs (MSICUs) and one 16-bed Neuro-ICU, in addition to other critical care units not included in the intervention (i.e. CCU, CSU, PICU, and NICU). The facility does not have an inpatient palliative care unit or an inpatient hospice unit.

### Sample size determination

Two prior studies of joint rounding models, Braus et al. [26] and Walker et al. [29] had study populations of approximately 200 patients. Walker et al. demonstrated a significant effect of the intervention on median ICU LOS (7 vs. 11 days, p < 0.001), whereas Braus et al. did not (4 vs. 5 days, p = 0.14). Given that the model in the current study did not prescreen for patients with palliative needs and so the observed effect would likely be smaller, we targeted a sample size of 400 patients—double the population of these prior studies. In consultation with an ICU administrator regarding typical ICU admission rates and average census, we estimated that two 6-month enrollment periods would likely yield at least 200 patients in each group.

### Participants

**Fig 1:**
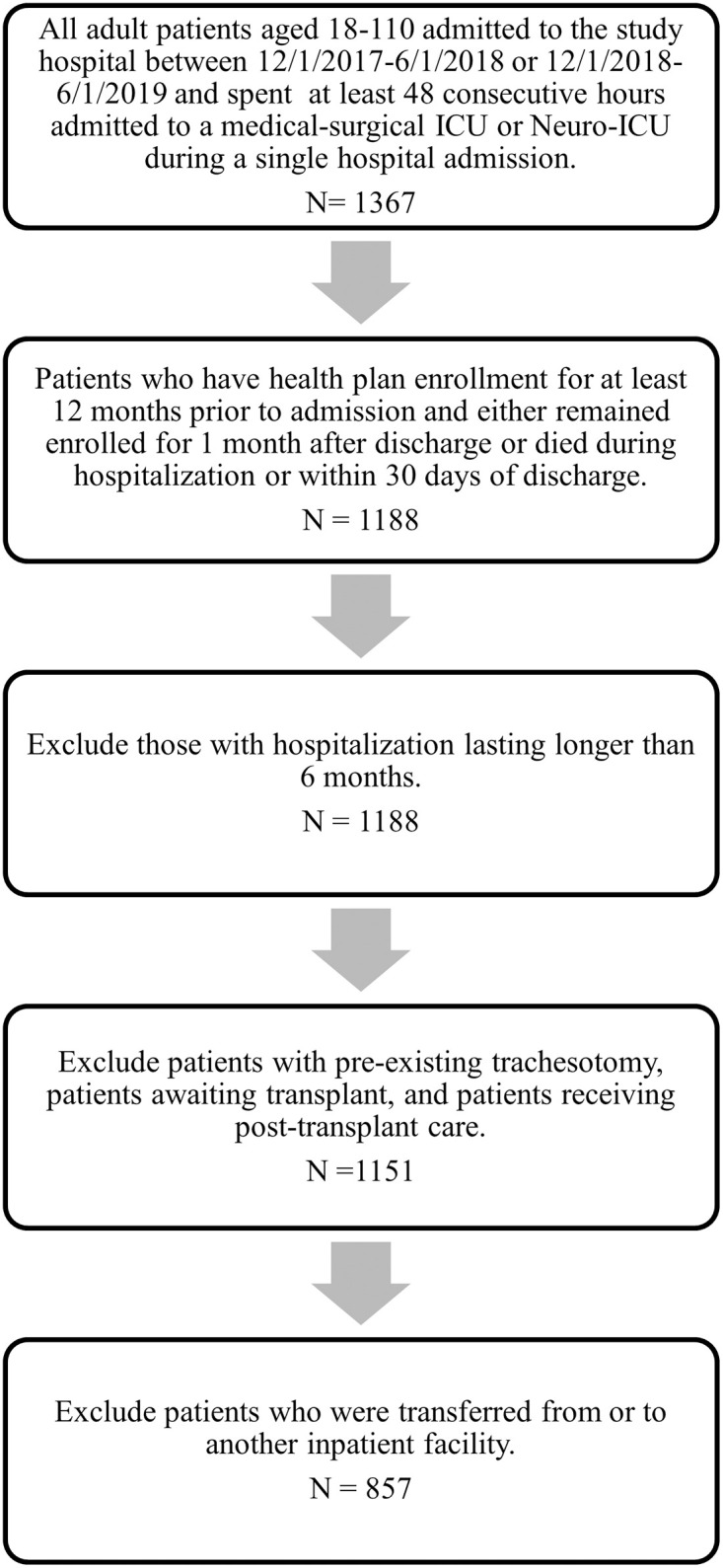
Cohort assembly.

Adults aged 18 and over who were admitted to either a medical-surgical ICU or the Neuro-ICU during the pre-intervention and post-intervention time periods were included regardless of diagnosis or reason for ICU admission. The pre-intervention control group included adult patients admitted during the 6-month period between December 1st 2017 and June 1st 2018 and the intervention group included patients admitted between December 1st 2018 and June 1st 2019. These dates were chosen to control for seasonal variation in illness patterns affecting ICU patient population. Patients were included only if they had spent at least 48 consecutive hours in ICU during their hospitalization, with the intention of excluding patients with only transient ICU needs (e.g. management of anaphylaxis or alcohol withdrawal). To ensure complete baseline and follow-up data, inclusion was restricted to patients who had active KP membership at least 1 year prior to hospitalization and at least 30 days after discharge. Patients were excluded if they had pre-existing tracheostomy, were awaiting organ transplant, or receiving post-transplant care, because such patients typically require prolonged ICU stay as part of appropriate routine care, which cannot be reasonably abbreviated based on improved communication alone. Patients were also excluded if they were hospitalized for more than 6 months in order to prevent crossover between groups, and if they had transferred from or to another inpatient facility, which would make it impossible to accurately calculate LOS (Fig 1).

### Intervention

There are two separate IPC teams rounding in the hospital on weekdays only, each of which includes a physician, a registered nurse, and a social worker with a shared chaplain between the two teams. Beginning in October of 2018, one of these two teams was designated to focus on palliative care consultations ordered for ICU patients. Concurrently, the physician on this team began joining the pre-established ICU multidisciplinary rounds 2–3 times per week (typically Tuesdays and Thursdays and sometimes Fridays). This multidisciplinary rounding team included an ICU physician, social worker, charge nurse, nursing case manager, infection control nurse, dietician, and a rehabilitation representative. This rounding team meets for approximately 60–90 minutes every morning and spends 2–5 minutes discussing each patient with the bedside nurse outside of the patients’ rooms in the 2 MSICUs and 1 Neuro-ICU. On the days in which the IPC physician joined these rounds, the physician would help identify patients who may benefit from IPC consultation, review updates on patients already followed by IPC, and provide advice on other ICU patients without formal consultation. When indicated, formal IPC consultation included: assessment of the patient’s decision-making capacity and symptom burden by one of the IPC team’s physicians or nurses, outreach to surrogate medical decision-maker(s) when appropriate, coordination of family meetings with appropriate medical team representation, and longitudinal support for as long as indicated. Indications for longitudinal follow-up included ongoing clarification of treatment goals, psychosocial and spiritual support, and symptom management.

As a result of participating regularly in the ICU multidisciplinary rounds, the IPC physician and team had increased interaction with the ICU physicians (including attending physicians, pulmonary and critical care fellows, and residents from multiple specialties) and non-physician staff. This allowed for impromptu case-based teaching of palliative care principles both in and out of rounds, which was not quantified in the current study.

### Data collection and statistical analysis

The KPSC Institutional Review Board approved this study on January 19th, 2021 and waived individual subject informed consent. All methods were performed in accordance with the relevant guidelines and regulations. All data were obtained from the KP Southern California Research Data Warehouse, which is an integrated and indexed repository of patient-related data from multiple contexts including the electronic medical record (EMR). The study’s statistician accessed and analyzed the data between February 5th and December 31st, 2021. All data were deidentified prior to analysis, and the author had access to identifying information solely when conducting data validation. The primary outcome measure was median ICU LOS, measured in days. Secondary outcome measures included median hospital LOS (in days), median number of days connected to a mechanical ventilator via endotracheal tube (ETT), mortality during hospitalization, emergency department (ED) and hospital bounceback within 30 days after discharge, and whether cardiopulmonary resuscitation (CPR) with chest compressions was performed during the hospitalization. CPR was identified in the EMR based on presence of a progress note describing a “code blue” event, and then the author performed a limited chart review of these notes to determine whether chest compressions were performed.

Baseline demographic data included age, sex, Charlson Comorbidity Index [45], race, preferred language, and whether IPC was consulted during admission. For patients with a preferred language other than English, need for language interpretation was included as a separate variable given that not all non-native English speakers require interpretation, and the need for interpretation can represent a significant barrier to communication independent of the native language. As such, need for interpretation was deemed a more useful independent variable for secondary analysis than language spoken. Prior palliative care involvement in the 12 months prior to admission was also included as a variable since prior involvement would denote a pre-existing chronic and serious illness in addition to any acute illness resulting in ICU admission. Prior palliative care involvement was defined as the presence or absence of either an inpatient note by a provider whose service was listed as either “hospice” or “palliative medicine” or an outpatient note by a provider whose department specialty was listed as either “continuing care” or “hospice care.”

Baseline characteristics and outcome measures were compared by Wilcoxon and chi-square test for continuous and categorical variables respectively. Negative binomial regression was used to analyze the primary outcome measure: number of days in ICU. SAS Enterprise Guide 7.1 (SAS Institute Inc., Cary, North Carolina) was used for data analysis. All statistical tests were two-sided. Point estimates and 95% CIs are presented.

## Results and discussion

During the combined study period 1,367 patients were identified with ≥48 hour stays in the MSICU or Neuro-ICU. 179 patients were excluded based on KP membership requirements, 37 were excluded due to transplant status or pre-existing tracheostomy, and 294 were excluded due to transfer to or from another acute medical care facility (Fig 1). In the end 857 patients were included in the study, 431 of whom were in the pre-intervention control group and 426 in the intervention group. Table 1 presents demographic data for the included patients. These groups did not differ significantly in age, sex, race/ethnicity, preferred language, Charlson Comorbidity Index, prior palliative care involvement, or IPC consultation during their hospitalization. The pre-intervention group was more likely to have documentation in their medical record noting a requirement for language interpretation (15.8% vs. 10.3%, p = 0.02).

**Table 1:** Demographic data.

	Pre-intervention	Intervention	Total	Significance
	N = 431	N = 426	N = 857	
**Age**, mean (SD)	65.5 (17.24)	64.2 (16.08)	64.9 (16.67)	p = 0.08
**Sex**				p = 0.13
**Female**	194 (45%)	214 (50.2%)	408 (47.6%)	
**Male**	237 (55%)	212 (49.8%)	449 (52.4%)	
**CCI**^a^ **Score**				p = 0.23
**0–1**	135 (31.3%)	155 (36.4%)	290 (33.8%)	
**2–4**	145 (33.6%)	141 (33.1%)	286 (33.4%)	
**≥5**	151 (35%)	130 (30.5%)	281 (32.8%)	
**Race/Ethnicity**				p = 0.73
**Black**	66 (15.3%)	69 (16.2%)	135 (15.8%)	
**White**	129 (29.9%)	129 (30.3%)	258 (30.1%)	
**Hispanic**	159 (36.9%)	143 (33.6%)	302 (35.2%)	
**Other**	77 (17.9%)	85 (20%)	162 (18.9%)	
**Prior Palliative Care** ^b^	24 (5.6%)	27 (6.3%)	51 (6%)	p = 0.63
**IPC**^c^ **Consultation**	60 (13.9%)	68 (16%)	128 (14.9%)	p = 0.40
**Preferred Language**				p = 0.31
**English**	350 (81.2%)	360 (84.5%)	710 (82.8%)	
**Spanish**	66 (15.3%)	50 (11.7%)	116 (13.5%)	
**Other**	15 (3.5%)	16 (3.8%)	31 (3.6%)	
**Interpreter Needed** ^d^	68 (15.8%)	44 (10.3%)	112 (13.1%)	**p = 0.02**

Except where indicated all values are reported as n (%).

^a^CCI: Charlson Comorbidity Index is a tool for predicting 1-year mortality based on the number and seriousness of comorbid conditions.

^b^Prior Palliative Care: patients with a documented inpatient or outpatient visit with a Palliative Care provider in the 12 months leading up to the hospital admission.

^c^IPC consultation: whether a consult order was placed in the EMR during the present admission.

^d^Interpreter Needed: patients with a preferred language other than English who have designated the need for language interpretation.

The univariate, unadjusted outcomes for the primary and secondary outcome measures are presented in Table 2. Patients in the intervention group spent fewer days in the ICU (3.7 days vs. 3.9 days, p = 0.05) and fewer total days in the hospital (7.5 days vs. 7.8 days, p < 0.01) when compared to the pre-intervention control group. Fewer patients received CPR in the intervention group compared to the pre-intervention control group, but this difference did not meet the threshold for statistical significance [13(3.1%) vs. 23(5.3%), p = 0.10]. The groups did not differ significantly in rate of hospital mortality, number of days connected to mechanical ventilation via endotracheal tube, or bounceback to the ED or hospital.

**Table 2:** Outcomes.

	Pre-intervention	Intervention	Total	Significance
	N = 431	N = 426	N = 857	
**Days in ICU**, median (IQR)	3.9 (3.3)	3.7 (3.2)	3.7 (3.2)	**p = 0.05**
**Days in hospital**, median (IQR)	7.8 (9.7)	7.5 (8.1)	7.6 (8.6)	**p < 0.01**
**Days on ETT**, median (IQR)	4.0 (6.5)	4.0 (6.0)	4.0 (6.0)	p = 0.48
**Death during hospitalization**	60 (13.9%)	59 (13.8%)	119 (13.9%)	p = 0.98
**30 Day ED bounceback**	87 (20.2%)	103 (24.2%)	190 (22.2%)	p = 0.16
**30 Day hospital bounceback**	30 (7%)	29 (6.8%)	59 (6.9%)	p = 0.93
**Received CPR** ^a^	23 (5.3%)	13 (3.1%)	36 (4.2%)	p = 0.10

Except where indicated all values are reported as n (%).

^a^Received CPR: patients who received CPR with chest compressions after cardiac arrest during the hospitalization.

Multivariable analysis (Table 3) of the primary outcome demonstrated that patients in the post-intervention group had shorter ICU LOS (RR 0.82, 95% CI 0.70–0.97, p = 0.02) and those with prior palliative care involvement had a significantly longer ICU LOS (RR 1.46, 95% CI 1.04–2.06, p = 0.03) when controlling for other variables. ICU LOS did not significantly correlate with age, male sex, race/ethnicity, need for interpretation, or CCI score.

**Table 3:** Multivariable analysis.

	Relative Risk (95% CI)	Significance
**Experimental group**	0.82 (0.7, 0.96)	**p = 0.02**
**Age** ^a^	1.01 (0.98, 1.04)	p = 0.44
**Male sex**	0.98 (0.83, 1.15)	p = 0.80
**Race/Ethnicity**		
**Black**	1.00 (0.78, 1.30)	p = 0.98
**Hispanic**	1.18 (0.95, 1.48)	p = 0.14
**Other**	1.23 (0.97, 1.56)	p = 0.09
**Prior Palliative Care**	1.46 (1.04, 2.06)	**p = 0.03**
**Interpreter Needed**	1.18 (0.90, 1.54)	p = 0.24
**CCI Score**		
**2–4**	0.90 (0.73, 1.11)	0.32
**≥5**	0.89 (0.72, 1.10)	0.29

^a^Age: patients were divided into age groups based on 5-year units (e.g. age 60–65)

## Discussion

Similar to prior studies [17, 24, 26, 28–32], the current study demonstrated that palliative care involvement in the care of ICU patients results in decreased ICU and hospital LOS, in this case utilizing a joint IPC-ICU multidisciplinary rounding model. However, the magnitude of change in median ICU LOS (3.7 days vs. 3.9 days) and median hospital LOS (7.5 days vs. 7.8 days), is arguably not clinically significant. The observed statistical significance may be attributable to the fact that our sample size was double the intended size and thus the study was overpowered.

Braus et al. [26] demonstrated that IPC representation during ICU rounds resulted in more frequent and timely family meetings and shorter hospital LOS for all ICU patients that met certain trigger criteria indicating high risk of mortality, morbidity, or unmet palliative care needs. In that study, ICU LOS was only shortened in the subset of patients who died in the hospital. As in our study, there was no significant difference in IPC consultation between the pre- and post-intervention groups, which suggests that any benefit of palliative care involvement during rounds is not solely dependent on increasing consultation rate. Where our study differs from the Braus et al. study is that we did not screen for patients who met trigger criteria, and thus looked more broadly at the ICU patient population. When we compare our overall IPC consultation rate of 14.9% to the 19% consultation rate in Braus et al.’s study, the lower rate suggests that our broader approach included a larger number of patients with no need for formal palliative consultation. Of note, however, the rate of consultation in the Braus et al. study was also low even despite screening for patients who appeared to have a palliative care need based on chart review. This may suggest that there is reluctance on the part of the ICU providers to formally consult palliative care despite both screening and palliative care representation during rounds. Another significant distinction between the studies is that the current study did not specifically measure the frequency or timeliness of family meetings; as such, we can only postulate that the observed differences are similarly mediated by improved communication between the care team and patients or their surrogate decision-makers.

With regard to secondary outcomes, implementation of the joint rounding model did not result in any change in patient bounceback rate to the ED or hospital, which had been demonstrated in prior research [25]. There was also no significant difference in the number of days patients received mechanical ventilation via an endotracheal tube (ETT) before proceeding with either extubation or tracheostomy. Of note, this study did not differentiate between “compassionate extubation” for refractory respiratory failure (resulting in death from respiratory failure) and medically indicated extubation based on clinical improvement. The duration of mechanical ventilation in patients who can be safely extubated is more reflective of medical necessity rather than the effectiveness of serious illness communication, and so we would not expect Palliative Care involvement to impact duration of ventilation in these patients.

The decreased rate of CPR in the post-intervention group compared to the pre-intervention seems clinically significant as it was almost halved. However, it did not meet criteria for statistical significance. This is likely due to the overall low incidence of CPR in the cohort, resulting in insufficient statistical power.

We might expect that prior palliative care involvement, either outpatient or in prior hospitalization(s), would result in quicker ICU turnaround time as a reflection of prior discussions around treatment goals and preferences surrounding life-sustaining treatments. Instead, the current study showed that prior palliative care involvement was associated with longer ICU LOS. The increased LOS likely reflects that this patient population is more medically complex at baseline, since palliative care is typically reserved for patients with chronic and progressive illnesses at more advanced stages. As a result, these patients may require more extensive care when they present acutely to the ICU.

### Limitations

The impact of palliative care interventions on qualitative outcomes such as quality of communication is difficult to capture in a quantitative study such as this. Like prior studies, we measured healthcare utilization outcomes, which may reflect improved illness communication but are also influenced by many other patient-related and institutional variables. Furthermore, this study was conducted after implementation of the joint IPC and ICU multidisciplinary rounding model, necessitating a retrospective design. As such we cannot infer a causal relationship between the intervention and the observed outcomes, and we can only infer that these outcomes may be related to a change in ICU culture with an emphasis on improved illness communication.

By design, our study did not focus on a subset of patients with pre-defined palliative care needs as defined by screening criteria, but rather on a broader population of MSICU and Neuro-ICU patients. Many patients admitted to ICU with straightforward diagnoses (e.g diabetic ketoacidosis) may not benefit from more formalized communication in the form of a family meeting. Additionally, many ICU patients do not require mechanical ventilation; of those who are intubated, many do not have refractory respiratory failure that would require the medical team to address the question of proceeding with either tracheostomy or compassionate extubation. Since we did not look at individual diagnoses or identify the presence or absence of refractory respiratory failure, secondary analysis of these variables was not done. Had we looked at a subset of the study population with refractory respiratory failure, we might have observed a difference in the duration of mechanical ventilation via ETT. Similarly, a population selected based on palliative care screening criteria might have shown more robust differences in the primary and secondary outcomes.

Zalenski et al. [32] and May et al. [31] demonstrated that timeliness of IPC consultation is a key contributing factor in the reductions of LOS and cost, respectively. The current intervention involved having an IPC physician join the pre-established ICU multidisciplinary rounds 2–3 times per week, typically Tuesdays and Thursdays and sometimes Fridays. The intermittent presence of IPC on these rounds means that the IPC physician may not learn about a large subset of patients until several days after ICU admission, or indeed he/she may miss certain cases altogether. Furthermore, the retrospective nature of the study did not allow for verification of the actual frequency of IPC presence during rounds. The study also did not measure the timeliness of IPC consultation (in those who received consultation) or the time to the first family meeting (as measured in Braus et al. [26]), so secondary analyses based on these measures was not possible.

This study was limited to the 2 MSICUs and 1 Neuro-ICU at a single tertiary Kaiser Permanente (KP) medical center. It is unclear whether the results presented are generalizable to other critical care units (e.g. Coronary Care Unit). Since this hospital is not a trauma center and thus receives trauma cases only after stabilization at other medical centers, trauma patients were not included in this study by nature of the design. The present study also focused only on patients with active KP insurance policies, which limits generalizability to patients in other non-HMO healthcare systems.

### Areas for future study

The current study used utilization metrics such as ICU and hospital LOS to reflect the quality of care and specifically communication in the ICU. There is a paucity of research looking at how ICU and IPC collaborative interventions such as this study’s joint rounding model might impact the perceived quality of communication and quality of care provided. A prospective study comparing patient/surrogate and provider attitudes about care and communication quality before and after implementing a joint rounding model would help demonstrate whether such collaboration is beneficial from a more qualitative standpoint.

Additionally, this study looked at several utilization outcomes that may impact healthcare costs, but did not measure cost per patient or overall ICU operational costs before and after the intervention. A follow-up cost-analysis study would help identify whether there is a financial benefit to the current joint rounding model in addition to any qualitative benefits.

Another future study dedicated to exploring the secondary outcome of days connected to mechanical ventilation via endotracheal tube could better define the impact of IPC in supporting the specific decision surrounding compassionate extubation. The current study found no significant difference in this outcome measure, likely because it included both patients who did not require intubation and those who had reversible respiratory failure allowing for safe extubation. A future study focused solely on patients with refractory respiratory failure may better demonstrate whether IPC and ICU collaboration affects the decision-making process around compassionate extubation vs. tracheostomy in these patients.

## Conclusions

The current study demonstrated that a joint IPC-ICU multidisciplinary rounding model is associated with statistically significant reduction in ICU and hospital LOS. The clinical significance is unclear, given the low magnitude of this reduction in LOS. The retrospective design of the study further limits our ability to determine how the intervention may have contributed to the observed outcomes. Further research is needed to determine the qualitative benefit of this type of intervention for patients, their families, and their treatment teams as well as the cost-effectiveness of this approach to palliative care in the ICU.

## Supporting information

S1 Checklist *PLOS ONE* clinical studies checklist. (DOCX)

## Abbreviations

CCI: Charlson Comorbidity Index
CCU: Cardiac Care Unit
CPR: Cardiopulmonary resuscitation
CSU: Cardiac Surgical Unit
DNR: Do Not Resuscitate
ED: Emergency Department
EMR: Electronic Medical Records
ETT: Endotracheal tube
ICU: Intensive Care Unit
IPC: Inpatient Palliative Care
KP: Kaiser Permanente
LOS: Length of Stay
MSICU: Medical-Surgical ICU
NICU: Neonatal ICU
PICU: Pediatric ICU
